# ‘Difficult Conversations with Patients’—A Modified Group Objective Structured Clinical Experience for Medical Students

**DOI:** 10.3390/ijerph18115772

**Published:** 2021-05-27

**Authors:** Piotr Przymuszała, Patrycja Marciniak-Stępak, Magdalena Cerbin-Koczorowska, Martyna Borowczyk, Katarzyna Cieślak, Lidia Szlanga, Łucja Zielińska-Tomczak, Ryszard Marciniak

**Affiliations:** 1 Department of Medical Education, Poznan University of Medical Sciences, 60-806 Poznan, Poland; pprzymuszala@ump.edu.pl (P.P.); zielinskatomczak@ump.edu.pl (Ł.Z.-T.); rmarcin@ump.edu.pl (R.M.); 2 Department of Medical Simulation, Poznan University of Medical Sciences, 60-806 Poznan, Poland; pmarciniak@ump.edu.pl (P.M.-S.); martyna.borowczyk@ump.edu.pl (M.B.); lszlanga@ump.edu.pl (L.S.); 3 Department of Pediatric Oncology, Hematology and Transplantology, Poznan University of Medical Sciences, 60-572 Poznan, Poland; 4 Medical Simulation Centre, Poznan University of Medical Sciences, 60-806 Poznan, Poland; kcieslak@ump.edu.pl

**Keywords:** group objective structured clinical experience, simulated patients, delivering bad news to patients, difficult conversations with patients, medical students

## Abstract

This study presents a modified Group Objective Structured Clinical Experience (GOSCE) focused on difficult conversations, in which, due to limited time and financial resources, only some students could actively participate in scenarios. We aimed to evaluate the intervention, including differences between them and observers. The intervention was organized for sixth-year medical students at a Polish medical university. The study protocol assumed a pre-post analysis of students’ attitudes and self-efficacy of communication skills and their opinions about the intervention. Complete questionnaire pairs were returned by 126 students. The pre-post analysis revealed a significant improvement in their self-efficacy levels of almost all skills as well as their affective attitudes and belief in outcomes of communication learning. The improvement was significant among both the active participants and observers. It also showed a decrease in the motivation score, significant only in females. Regardless of their roles, students had positive opinions about the course and its particular aspects. The modified GOSCE may be an enjoyable and effective learning experience for students, especially in the light of limited resources. However, changes in their motivation score suggest the necessity to increase the importance of communication learning in the curriculum.

## 1. Introduction

Giving bad news to patients is considered one of the most challenging and stressful experiences in physicians’ professional life [1]. The fear and anxiety associated with them may also negatively affect patients leading to doctors delaying or avoiding difficult conversations or the hit-and-run delivery [1,2]. Meanwhile, the literature describes many advantages and possibilities related to the use of simulated patients in the training of medical students. They are highly rated by students and medical teachers in terms of the authenticity and emotional depth of their performance [3,4]. They also offer an unprecedented level of standardization of students’ experiences compared to the traditional bedside teaching model [5]. Moreover, the learning experience does not cause inconvenience or harm to real patients [6,7]. As a result, simulated patients allow medical students to re-enact even the most challenging scenarios, including giving bad news to patients or their families, in safe and less-stressful conditions. Interactions with simulated patients can boost students’ confidence and self-esteem, increasing their comfort while talking to patients in the future [8]. Simulated patients can also provide students with constructive feedback and positive reinforcement on their performance, which can additionally influence their abilities and reduce the stress associated with future contact with patients [6,7,8,9,10].

However, the use of simulated patients may be limited due to the considerable amount of time and financial resources this method requires [9,11]. They can be a barrier, especially in countries with limited resources, where relatively cheaper non-experiential learning methods can be favored, for instance, in Poland. Meanwhile, a GOSCE (Group Objective Structured Clinical Experience) is a learning method in which students move through scenarios in groups, allowing them to observe and learn from their colleagues’ performance. Studies published so far showed that this method increases students’ self-efficacy and attitudes towards communication skills learning [12,13]. Ludwig et al. [12] showed that while, in the opinion of students, the formative GOSCE is an enjoyable and effective experience, it may serve as a more time- and resource-friendly substitute for their individual interactions with simulated patients. In their study, the GOSCE was conducted for third-year medical students during their Internal Medicine Clerkship. Groups consisting of 4–6 students accompanied by one faculty member rotated through four scenarios pertaining to counseling on quitting smoking, speaking with an angry patient, discussion of treatment options with a diabetic patent, and informing a patient about the cancer diagnosis. In a pre-post analysis of students’ confidence in general, case-specific, and group clinical communication, statistically significant improvements were found, with the greatest change observed in regard to talking with an angry patient. Additionally, students’ evaluation of the course showed very positive responses, with more than 90% of them agreeing that the GOSCE taught them something new and around 65% being more comfortable with giving and receiving feedback and group work. Due to its low stakes nature, the authors recommended it for practicing giving bad news to patients. In a study by Konopasek et al. [13], the GOSCE was organized for third- and fourth-year medical students during their Medicine and Pediatrics clerkships. Students were divided into groups of three to five, and scenarios involved communication skills and clinical reasoning exercises with diverse emotional responses from simulated patients, including distress about pain, anxiety, anger, unexpected calm, optimism, and denial. At the end of the GOSCE, students were asked to fill a retrospective pre-post questionnaire on their self-efficacy and attitudes towards communication learning. The results obtained by them showed significantly higher scores after the training than before. Students also very highly rated feedback from all sources, namely other students, simulated patients, and faculty members. The GOSCE was also successfully implemented as a formative assessment by Sulaiman et al. [14] for medical students of the first, second, and third years. The stations included a range of clinical skills to be performed by students, for instance, history taking, physical examination, explanation skills, basic medical procedures, and data interpretation. After the GOSCE, students and the faculty were asked to complete a survey on their opinions. The majority of students believed that the received feedback was informative, and they favored small group feedback over the individual one. Similarly, the formative GOSCE format was also preferred by the faculty members. The experience was seen in both parts as a knowledge and skills sharing opportunity for students [14]. Additionally, observing others may strengthen students’ self-assessment ability and help them acknowledge their own mistakes [12]. 

In this study, a modified version of GOSCE is presented in the context of difficult conversations with patients. Contrary to previous studies, students did not rotate between the scenarios in smaller groups. Instead, at one time, only one scenario was re-enacted, and students not involved in it observed the scene together with the faculty from the debriefing room via cameras. This was due to limited human and financial resources and the amount of time dedicated to the course in the curriculum. As a result, we were not able to provide all students with the possibility to actively participate in a scenario. Although similar cases, when not all students had the opportunity to be actively involved, were reported previously [13], the differences between both groups of students were not examined. Moreover, the literature mostly describes the GOSCE from the third or fourth-year medical students’ perspective, and our study focused on sixth-year medical students. In light of the decline of students’ communication skills, attitudes towards them, and empathy levels with time [15,16,17,18,19], this may constitute another factor influencing the evaluation of the intervention.

We aimed to evaluate the modified version of GOSCE in terms of its influence on students’ attitudes towards communication skills and belief in their capabilities. The second aim was to collect students’ opinions on the intervention, including its usefulness and attractiveness. For this purpose, both closed and open questions were used. Finally, given that only some students had the opportunity to be actively involved in the scenarios due to the aforementioned financial and curriculum constraints, we also decided to assess potential differences in students’ responses regarding this factor.

## 2. Materials and Methods

### 2.1. Study Settings

‘Difficult conversations with patients’ were introduced in the academic year 2019/2020 as part of the 1-week Advanced Medical Simulation clerkship (AMSC) for sixth-year medical students of our University. The 4 h course was conducted on the fourth day of the AMSC and focused on communication skills development with particular emphasis on difficult conversations, including delivering bad news to patients or the family. Approximately 12–14 students participated in each edition of the course (depending on group sizes imposed by the Dean’s Office). They were supervised by a team consisting of at least one physician and a psychologist. The first hour of the course was dedicated to introducing students to the topic and familiarizing them with the EMPATHY protocol as a valuable tool during difficult conversations [20]. Then students were presented with three scenarios.

The first of them involved a case of a young woman, a professional equestrian, who fell off the horse. As a result, her leg was crushed and had to be amputated. The student’s task was to take the history, inform the patient about the unfavorable diagnosis, and collect the informed consent for the procedure.

In the second case, students met with the wife of a patient, who unexpectedly passed away the night before due to the consequences of chronic liver failure in the context of alcoholic cirrhosis. Their task was to inform the wife about the death of her husband. During the conversation, the wife at first disbelieves and denies the news. She inquires further into the causes of death. Then she starts crying and partially blames herself, admitting that she used to wish for her husband’s death when he was drinking heavily. She also mentions her difficult financial situation and that her husband left her with serious debts.

The third scenario was partially the continuation of the second one. The student walks into the consultation room to meet with a colleague who shows clear signs of depression and resignation. Upon investigation, the student learns that the doctor is struggling with the unexpected death of the patient. The doctor blames him or herself for ‘killing the patient’ and plans to quit medicine to prevent future tragedies. The student’s role was to support and comfort the colleague in distress.

Consultations with simulated patients were conducted in a separate room. Every scenario involved one student who actively participated in it and two students observing it in the consultation room from the perspective of verbal and non-verbal aspects of communication. The remaining students watched the scene from the debriefing room via cameras. Henceforth active participants of any of the scenarios will be denoted as ‘doctors’ and remaining students as ‘observers.’ After each scenario, a debriefing session was carried out according to the Pendleton model [21]. After that, the next scenario was re-enacted with a different student in the physician role.

The study protocol involved a pre-post analysis of changes in students’ self-efficacy and attitudes towards communication skills learning. They were approached on the first day of the AMSC and asked to fill a paper version of the first questionnaire. The second questionnaire was completed on the fourth day immediately after the course. The initial research plan intended to invite all 271 sixth-year medical students of the academic year 2019/2020 to participate in the study. However, it was disrupted by the decision of Polish government authorities to cancel all traditional face-to-face classes at schools and universities due to the coronavirus disease 2019 (COVID-19) pandemic outbreak in March 2020. Consequently, only students taking the course before the lockdown participated in the study. Altogether, 11 editions of the course were fully completed before the lockdown. Additionally, two groups started their AMSC in the week when the lockdown was announced. Since the decision had an immediate effect and was proclaimed before their scheduled course date, they could not participate in it and fill the second survey.

### 2.2. Participants of the Study

Of 172 students participating in the AMSC before the COVID-19 pandemic outbreak in March 2020, 166 students returned the first questionnaire, and 126 students completed the second questionnaire. Among the 40 missing questionnaires, 13 students resigned from further participation in the study, and 27 were affected by the decision of the Polish government to cancel all face-to-face classes at universities that was announced in the meantime. Among 126 students who completed both forms, 80 (63.5%) were female and 46 (36.5%) were male. There were also 30 (23.8%) doctors and 96 (76.2%) observers. The age of respondents ranged from 22 to 33 (mean = 24.6; median = 24; interquartile range = 24–25).

### 2.3. Research Tools

**Figure 1:**
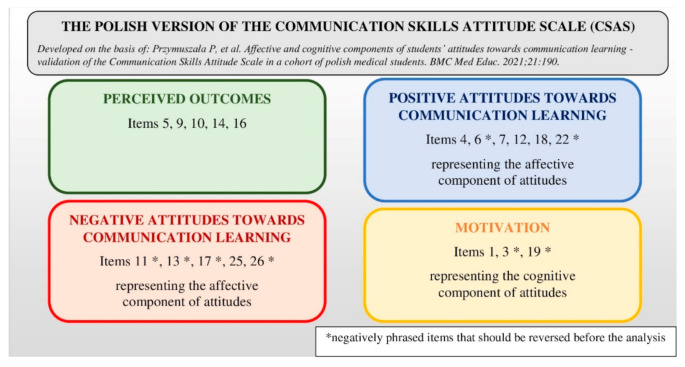
Visualization of the Polish version of the Communication Skills Attitude Scale.

Both questionnaires used in the study were drafted by the first author and presented to a panel of three experts in medical education and communication skills training for review. They were also pre-tested on a sample of 10 students from the first edition of the clerkship in terms of their understandability. Both the first and second questionnaire had a similar layout and intended to capture a broad range of quantitative and qualitative data. They started with the validated Polish version of the Communication Skills Attitude Scale (CSAS) to capture changes in students’ attitudes before and after the course [22]. The Polish version of CSAS was translated and adapted based on the original English version [23]. It consists of four subscales evaluating perceived outcomes of communication learning, positive and negative attitudes towards it, and factors motivating students to learn communication. Items are scored from 1 (strongly disagree) to 5 (strongly agree). A visualization of the Polish version of the CSAS is presented in Figure 1. The next section of both questionnaires involved the assessment of students’ self-efficacy of their particular communicational abilities. Questions were scored on a 5-point Likert scale from 1 (very poor) to 5 (very good). The second questionnaire also contained closed and open questions on students’ opinions about the intervention and ratings of its particular aspects. Both questionnaires were anonymous. However, for the purpose of the study, students were asked to disclose whether they participated as doctors or observers. They were also asked about their age and gender.

### 2.4. Data Analysis

Data obtained from the questionnaires were transcribed and subjected to subsequent analysis. Due to the fact that participation in the study was voluntary, and students had no obligation to complete the questionnaires, some of them filled only one questionnaire. Given the aim of the study, in such cases, incomplete sets of data were excluded from further analysis. Quantitative data were analyzed with the Wilcoxon signed-rank test and the Mann–Whitney U test, as appropriate, using the Statistica software (StatSoft) (version 13.3) (TIBCO Software Inc., Palo Alto, Santa Clara, CA, USA). Qualitative data were analyzed by two independent researchers.

### 2.5. Ethical Considerations

Prior to the study, its protocol was presented to the institutional Bioethics Committee, which, under the Polish legal system, waived the necessity for ethical approval as the study was not a medical experiment and did not involve patients (Case number: KB nr 946/19). Additionally, we made efforts to comply with the Ethical Guidelines for Educational Research [24]. Before participating in the study, students were informed about its aims and protocol. They were also assured about its voluntary and anonymous character. Students who agreed to participate in the study were also asked to sign a written consent form. The document contained the information above and explicitly emphasized that if they decided not to participate in the study or resigned from further participation, it would have no negative consequences for them.

## 3. Results

### 3.1. Students’ Attitudes towards Communication Learning

As presented in Table 1, a pre-post analysis of the CSAS subscales revealed a significant increase in students’ positive attitudes towards communication skills learning and their belief in its perceived outcomes with a significant reduction of their negative attitudes in all analyzed subpopulations. We also observed a decrease in their motivation scores, but it was significant only in the case of female students. The differences between pre- and post-intervention scores in regard to participants’ gender and the role they played during scenarios were not significant in any of the subscales.

### 3.2. Students’ Self-Efficacy of Their Communication Skills before and after the Course

**Figure 2:**
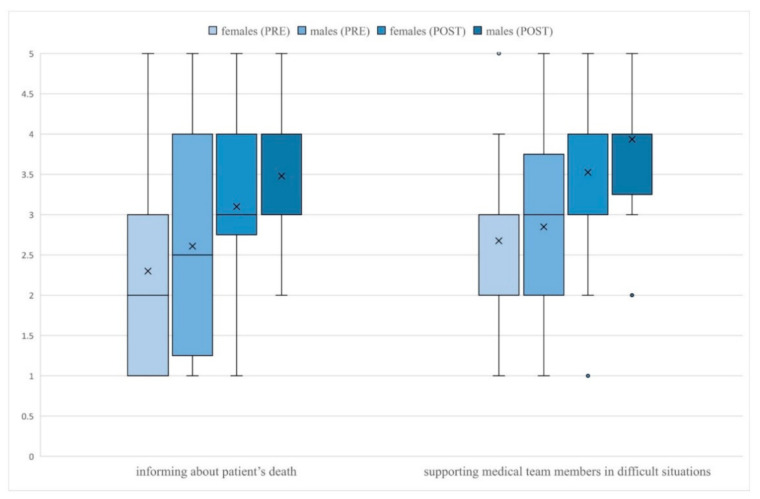
Gender differences in students’ self-efficacy ratings.

**Figure 3:**
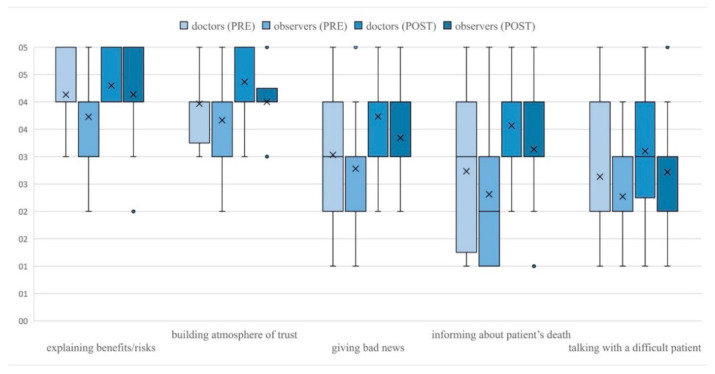
Differences in ratings of doctors and observers.

Students rated their communication skills after the course significantly higher in regard to nearly all skills (Table 2). After the intervention, male students rated significantly higher than females their ability to inform the family about patient’s death (*p* = 0.029) and support members of the medical team in difficult situations (*p* = 0.020) (Figure 2). No other gender differences in students’ self-efficacy ratings were observed before and after the study. Before the intervention, doctors gave significantly higher ratings than observers only to their ability to explain the benefits and risks of a given procedure (*p* = 0.019). After the course, the difference between both groups was no longer statistically significant (*p* = 0.424). After the study, doctors rated their abilities significantly higher than observers in respect to building the atmosphere of trust (*p* = 0.017), giving bad news to patients or family (*p* = 0.032), informing the family about the patient’s death (*p* = 0.014), and talking with a difficult, demanding patient (*p* = 0.049) (Figure 3). Despite these differences, the improvement of pre-post self-efficacy ratings among observers was still statistically significant—building the atmosphere of trust (*p* < 0.001), giving bad news to patients or their family (*p* < 0.001), informing the family about the patient’s death (*p* < 0.001) and talking with a difficult, demanding patient (*p* < 0.001). No other significant differences in ratings of doctors and observers were noticed.

### 3.3. Students’ Opinions about the Course

The general impression from the course, its usefulness, and other aspects were rated as good or very good by the vast majority of participants (Table 3). Although observing others received the lowest rating among other aspects, it was still assessed as good or very good by more than three-quarters of participants. Students valued feedback from their teachers, simulated patients, and colleagues. However, feedback from teachers was rated significantly higher than that from simulated patients (*p* < 0.001) and other students (*p* < 0.001). There was no difference in ratings of feedback from simulated patients and students (*p* = 0.152). No differences in terms of students’ gender were observed in their assessment of the course and its aspects. In regard to the role, doctors rated significantly higher the atmosphere during the classes and their own engagement during the course. No other significant differences were noted.

The positive reception of the intervention is also visible in students’ agreement with statements describing the course (Table 4). Statistical analysis revealed no significant differences in students’ agreement levels regarding their gender and whether they were doctors or observers during scenarios. As presented in Table 5, most students in the physician role reported the reality of the experience and the motivation to help the patient. They generally appreciated the possibility of observing others and feedback received on their performance from simulated patients and other students. Most of them were not concerned with being watched by other students.

Finally, students were asked to answer open questions on their opinions about the course. Among its advantages and things they particularly liked about the intervention, students listed the possibility to practice communication and soft skills in a low-stakes encounter. They appreciated the reality of the experience combined with safe and controlled conditions and a simultaneous lack of responsibility. It allowed them to feel like real doctors in scenarios and consequently face new challenges, test themselves in stressful situations, and better prepare for future professional work. Students were impressed by simulated patients’ acting skills and the multisource feedback received during the course, combined with the opportunity to observe others and learn patients’ perspectives on their performance. In their opinion, the scenarios used were engaging and realistic. They enjoyed the atmosphere during the course and believed it was well planned. Students were especially pleased that difficult conversations with patients were finally brought up during their studies. They valued the knowledge and skills acquired during the course.

“*They allow us to face situations we didn’t have the opportunity to experience during classes in a hospital.”*

“*The possibility to test ourselves in safe conditions with difficult situations we may encounter in the future. Also, our reactions to them and the emotions they trigger.”*

*“The possibility to discuss the scenes afterward, having a sort of catharsis from emotions occurring during the scenes.”*

Among the disadvantages and things they did not like about the course, students mostly mentioned time and curriculum restrictions. They regretted they could not have more similar classes and participate in more scenarios. Regarding the course itself, some students explicitly emphasized that they did not notice any shortcomings. However, for some, the experience was not as real as they expected. Others disclosed that the discussion after some scenarios was too long for them. They suggested making it shorter to fit more scenes. Some students also admitted that they did not enjoy the perspective of being observed and observing others. In their opinion, it made the experience more stressful and less realistic. Finally, students disliked the fact that some of their colleagues did not show interest and engagement during the course.

“*Too little time for every student to participate in their own case.”*

“*The awareness that this is a simulation makes them a little unreal.”*

Students were also asked if some changes should be introduced to make the course better. In response, they expressed a strong demand to increase the amount of time dedicated to learning communication skills in the curriculum. They regretted that not every student had the opportunity to be in the physician role. Due to time limitations, they also suggested making the discussion after each scenario shorter and increasing the number of interactions with simulated patients.

“*During the course nothing, there should be more of them.”*

## 4. Discussion

In this pilot study, we presented a modified GOSCE introduced at our institution. The topic and scenarios used during the course involved difficult conversations. Although due to the limitations imposed by the COVID-19 pandemic we were not able to administer the survey to all sixth-year students as we had initially planned, the collected data show that the presented intervention was positively rated by students. Moreover, we observed a significant improvement in students’ attitudes towards communication skills and self-efficacy of their abilities. We also showed its positive effect on students who were not actively involved in the scenarios.

The proposed intervention positively influenced students’ affective attitudes towards communication learning and belief in its perceived outcomes. Meanwhile, students’ attitudes seem to be an essential precursor of the learning process and predictor of their future communication behaviors [25]. Konopasek et al. [13] also demonstrated a significant improvement in participants’ attitudes after the GOSCE experience. However, in their case, a retrospective pre-post-training questionnaire was used, which could potentially affect learners’ responses. Moreover, their study protocol did not use a validated tool to capture students’ attitudes. We used a validated Polish CSAS [22], and the questionnaires were distributed separately before and after the course within four days of each other. This allowed us to follow the phenomenon more thoroughly and reduce the risk of potential bias. With the retrospective pre-post testing or short intervals between the administration of questionnaires (e.g., on the same day), there is a risk that students may use their previous answers to modify their post-intervention ratings. However, when the interval is too long, other factors may affect students’ responses.

Interestingly, our results showed a decline in students’ scores in the ‘Motivation’ subscale. Although it was statistically significant only in female students, the tendency was present in all examined subpopulations. It may be partially connected with their increased self-efficacy after the intervention. For instance, Rees et al. [26] observed that individuals confident in their communication skills held more negative attitudes towards their learning. On the other hand, participation in the course could make students realize that the emphasis on communication skills in their curriculum is still low. Students often brought up this issue in response to open questions. The contrast between the big demand for communication classes expressed by them and the actual amount of time dedicated to it may act as a hidden curriculum lowering their motivation for learning.

Students’ self-efficacy of their communication skills also significantly increased as a result of the intervention. Brown et al. [27] showed that even a short intervention could positively influence students’ self-assessment of their communication skills. Similar results in the context of GOSCE were also observed previously by other researchers [12,13]. However, in this study, we presented a modified GOSCE with fewer participants actively involved in scenarios. As a result, we additionally decided to assess potential differences between active participants and observers. We noticed some significant differences between the post-intervention self-efficacy of students in the physician role and observers, mostly concerning difficult conversations with patients. At the same time, the improvement of observers’ confidence was still statistically significant, proving the role of other factors in the learning process during the GOSCE experience besides active participation in the scenario. Previous studies suggested that by observing the performance of colleagues and giving them feedback, students can more closely visualize an ideal approach to the situation and gain more insights into their own skills [12]. Our results seem to support this view, demonstrating that students’ participation in the communication course, even as observers, can improve their confidence in their abilities. The possibility to confront one’s views and observations with the feedback and tips from teachers, simulated patients, and other students should also be mentioned in this regard [12].

The majority of students positively rated the attractiveness and usefulness of the course and its aspects, which is mirrored by the results of other studies dedicated to the GOSCE [12,13]. They also noticed many positive outcomes of the course and believed that similar classes should be organized more often. The experience was enjoyable and meaningful for both active students and observers. Although observing others was rated the lowest among various aspects of the course, it was still appreciated by more than three-quarters of the participants. Meanwhile, as mentioned above, the possibility to watch other students and give them feedback is regarded as one of the major assets of the GOSCE [12]. The perception of observing other students did not differ significantly between doctors and observers. Moreover, most students in the physician role stated that the presence of other students did not bother them, and observing other students was an additional occasion to learn. However, around one-fifth of students seemed to think differently and associated being observed with additional stress and lower realism of the situation. A similar finding was reported by Sulaiman et al. [14], who noticed that some students prefer not to be observed and receive feedback individually out of embarrassment, among other causes. In their opinion, it may also be associated with students’ confidence in their skills. Another interesting finding of our study is that although students appreciated the feedback from all sources, there was a statistically significant difference between their ratings of the feedback from teachers and simulated patients or other students. By contrast, Konopasek et al. [13] found no differences in students’ ratings of feedback in regards to its source. Meanwhile, although simulated patients usually lack clinical experience and their feedback is rather subjective and focused on their personal feelings [28], no significant differences were found between them and medical teachers in evaluating students’ empathy and communication skills [29]. Furthermore, other students’ feedback might be perceived as less threatening than when it comes from teachers [12].

Several limitations of the presented pilot study should be acknowledged. Firstly, the described intervention was evaluated in a single-center study on one year of Polish medical students. Further studies are needed to evaluate the modified GOSCE in other populations, especially in medical education systems where occasions for communication training with simulated patients are still limited due to financial and time restrictions. Secondly, the COVID-19 pandemic outbreak prevented us from realizing the initial research plan and collecting data from all sixth-year students. Nonetheless, by the time the Polish government had proclaimed the closure of universities, we managed to collect pre-post responses from 126 students. Also, the number of male participants in the study was lower than females. However, among all 271 students, 176 (64.94%) were female and 95 (35.06%) male, so the proportions in this study seem to represent the general demographic characteristic of the sixth-year medical students in that year. Another limitation is that we were not able to introduce a control group within the study. However, the university’s internal regulations and Polish law do not allow different versions of a particular course to be designed and implemented for students of the same year. Therefore, it is difficult to ensure that our results are an effect of the course. Nonetheless, we planned the study to at least partially mitigate this risk. The pre-post interval was kept relatively short to minimize the influence of other factors on the results. During that period, students had no other clerkships. Additionally, medical students in our institution have no electives in the sixth year. Therefore, the potential effect of other factors influencing the self-efficacy and attitudes of students on study results seems smaller. Next, the research tools used in the study only measured changes in attitudes and self-efficacy of students, and we did not evaluate the impact of the intervention on their actual communication skills. This was caused by the limited human and financial resources and the amount of time dedicated to the course in the curriculum. As a result, we would not be able to conduct a series of pre- and post-intervention assessments of students’ communication skills with simulated patients, for instance. However, given the promising results of this pilot study and increasing emphasis on communication skills training in our university, we plan to continue our research after the COVID-19 pandemic. We will make efforts to conduct a study evaluating changes in students’ communication skills after the GOSCE. Organizing it as an elective for a smaller group of students should allow us to provide its participants with more scenarios and plan the control group among other students. Finally, the described course was taught by four of the authors of this study, which could have caused bias both in students’ opinions on the course and the process of data analysis. In order to prevent it, students were instructed and encouraged to give their true opinions, including negative ones. It should be again emphasized that their participation was completely anonymous and voluntary. Furthermore, in order to minimize the bias related to the data analysis process, four additional researchers were involved who did not participate in the described intervention.

## 5. Conclusions

To conclude, this study evaluated a modified GOSCE introduced at our institution. Due to financial, time, and curriculum restrictions, it differed from previously described interventions as it involved fewer actively participating students. Nonetheless, the experience and its aspects received very positive ratings from the students. It also significantly improved the self-efficacy of communication skills, affective attitudes towards learning them, and belief in its outcomes of both active participants and observers. Given the pre-post analysis of their motivation for communication learning constituting the cognitive attitudes, efforts should be made to increase the emphasis and frequency of communication training in the medical curriculum.

## Figures and Tables

**Table 1:** The comparison of the CSAS subscales scores before and after the intervention.

Subscale of the Polish Version of CSAS ^1^		*n*	Mean (SD)		*p*-Value ^2^
			PRE	POST	
Perceived outcomes	Total	126	4.06 (0.66)	4.28 (0.73)	<0.001
	Females	80	4.14 (0.63)	4.32 (0.63)	<0.001
	Males	46	3.93 (0.71)	4.23 (0.89)	0.002
	Doctors	30	4.13 (0.72)	4.42 (0.61)	0.004
	Observers	96	4.04 (0.65)	4.24 (0.76)	<0.001
Positive Attitudes Towards Communication Learning	Total	126	3.40 (0.63)	3.60 (0.65)	<0.001
	Females	80	3.39 (0.63)	3.55 (0.62)	0.004
	Males	46	3.41 (0.63)	3.68 (0.70)	<0.001
	Doctors	30	3.56 (0.61)	3.78 (0.58)	0.004
	Observers	96	3.35 (0.63)	3.54 (0.66)	<0.001
Negative Attitudes Towards Communication Learning	Total	126	3.93 (0.50)	4.14 (0.54)	<0.001
	Females	80	3.94 (0.49)	4.14 (0.57)	<0.001
	Males	46	3.90 (0.52)	4.14 (0.50)	<0.001
	Doctors	30	3.89 (0.54)	4.07 (0.55)	0.019
	Observers	96	3.94 (0.49)	4.16 (0.54)	<0.001
Motivation	Total	126	3.49 (0.81)	3.38 (0.88)	0.104
	Females	80	3.57 (0.75)	3.40 (0.80)	0.023
	Males	46	3.36 (0.89)	3.34 (1.02)	0.967
	Doctors	30	3.58 (0.84)	3.30 (0.84)	0.088
	Observers	96	3.47 (0.80)	3.40 (0.90)	0.309

^1^ CSAS—Communication Skills Attitude Scale; ^2^ *p*-values significant at level of 0.05.

**Table 2:** The comparison of students’ self-efficacy ratings before and after the course.

Students’ Self-Efficacy Ratings before and after the Course (1 = Very Poor; 5 = Very Good)		*n*	Mean (SD)	M	Q1	Q3	*p*-Value ^1^
verbal communication	PRE	126	3.75 (0.78)	4	3	4	0.014
	POST	126	3.92 (0.67)	4	4	4	
non-verbal communication	PRE	126	3.51 (0.86)	4	3	4	<0.001
	POST	126	3.75 (0.76)	4	3	4	
talking with an adult patient	PRE	126	3.95 (0.65)	4	4	4	0.066
	POST	125	4.05 (0.62)	4	4	4	
using language understandable to patients	PRE	125	3.83 (0.91)	4	3	4	0.076
	POST	126	3.98 (0.77)	4	3	5	
identifying patient’s needs, expectations	PRE	126	3.52 (0.81)	4	3	4	<0.001
	POST	126	3.80 (0.76)	4	3	4	
meeting patient’s needs, expectations	PRE	126	3.65 (0.74)	4	3	4	0.017
	POST	126	3.83 (0.76)	4	3	4	
identifying the patient’s emotions	PRE	126	4.00 (0.84)	4	4	5	0.043
	POST	126	4.16 (0.71)	4	4	5	
adequately reacting to patient’s emotions	PRE	126	3.36 (0.92)	3	3	4	0.005
	POST	124	3.63 (0.80)	4	3	4	
adjusting the conversation to patient’s capabilities and emotional state	PRE	125	3.51 (0.95)	3	3	4	<0.001
	POST	126	3.93 (0.76)	4	3	4	
verifying whether the patient understood provided information	PRE	126	3.60 (0.87)	4	3	4	<0.001
	POST	126	4.15 (0.74)	4	4	5	
showing patient respect and empathy	PRE	126	4.29 (0.76)	4	4	5	0.002
	POST	126	4.48 (0.56)	5	4	5	
obtaining informed consent from patients	PRE	126	4.02 (0.75)	4	4	4	0.005
	POST	126	4.24 (0.66)	4	4	5	
explaining benefits and risks of a given procedure	PRE	126	3.83 (0.75)	4	3	4	<0.001
	POST	126	4.17 (0.69)	4	4	5	
building the atmosphere of trust	PRE	126	3.74 (0.77)	4	3	4	<0.001
	POST	126	4.09 (0.73)	4	4	5	
giving bad news to patients or their family	PRE	126	2.84 (1.06)	3	2	4	<0.001
	POST	126	3.44 (0.85)	4	3	4	
informing the family about the patient’s death	PRE	126	2.41 (1.15)	2	1	3	<0.001
	POST	126	3.24 (0.87)	3	3	4	
talking with a difficult, demanding patient	PRE	126	2.36 (1.02)	2	2	3	<0.001
	POST	126	2.81 (0.90)	3	2	3	
supporting members of the medical team in difficult situations	PRE	126	2.74 (1.02)	3	2	3	<0.001
	POST	126	3.67 (0.88)	4	3	4	

^1^ *p*-values significant at level of 0.05; M—median; Q1—lower quartile; Q3—upper quartile.

**Table 3:** Students’ ratings of particular aspects of the course.

Rated Aspect of the Course (1 = Very Poor; 5 = Very Good)	*n*	Mean (SD)	M	Q1	Q3	Good or Very Good
General impression from the course	126	4.53 (0.69)	5	4	5	92.86%
Usefulness in communication skills learning	126	4.56 (0.72)	5	4	5	92.06%
Atmosphere during the course	126	4.78 (0.52)	5	5	5	98.41%
Teachers conducting the course	126	4.83 (0.39)	5	5	5	99.21%
The way SPs portrayed their roles	126	4.69 (0.58)	5	4	5	96.83%
Layout and equipment of the consultation room	126	4.71 (0.56)	5	5	5	94.44%
Scenarios used during the course	126	4.62 (0.62)	5	4	5	92.86%
Observing other students	126	4.22 (1.10)	5	4	5	77.78%
Feedback received from teachers	126	4.78 (0.49)	5	5	5	96.83%
Feedback received from SPs	126	4.52 (0.70)	5	4	5	91.27%
Feedback received from other students	126	4.41 (0.81)	5	4	5	88.89%
Own engagement during the course	126	4.29 (0.76)	4	4	5	87.30%
Engagement of other students during the course	126	4.25 (0.69)	4	4	5	88.89%

*n*—number of respondents; M—median; Q1—lower quartile; Q3—upper quartile; SPs—simulated patients.

**Table 4:** Students’ levels of agreement with statements describing the course.

Statement Describing the Course (1 = Definitely Disagree; 5 = Definitely Agree)	*n*	Mean (SD)	M	Q1	Q3	Agree or Definitely Agree
Classes with simulated patients are a good idea and should be organized more often.	126	4.53 (0.81)	5	4	5	89.68%
My communication skills improved after the course with simulated patients.	126	4.25 (0.83)	4	4	5	84.13%
The knowledge and skills from the course will be useful in my future professional carrier.	125	4.52 (0.74)	5	4	5	91.20%
After the course, it will be easier for me to talk with real patients.	126	4.37 (0.81)	5	4	5	86.51%
After the course, I have more appreciation for the significance of communication skills in the physician’s profession.	126	4.32 (0.93)	5	4	5	80.95%
Simulated patients were well-prepared and credible while playing their roles.	126	4.63 (0.57)	5	4	5	95.24%
The course with simulated patients constituted an interesting experience for me.	126	4.52 (0.73)	5	4	5	92.86%
Scenarios involved situations that can happen to me in my future work.	126	4.63 (0.60)	5	4	5	97.62%
Thanks to observing other students, I can better see my earlier mistakes.	126	4.12 (0.92)	4	4	5	78.57%
I think that I gained a lot as a result of participating in the course.	124	4.42 (0.73)	5	4	5	88.71%
I would willingly participate in a similar course in the future.	126	4.44 (0.94)	5	4	5	86.51%

*n*—number of respondents; M—median; Q1—lower quartile; Q3—upper quartile.

**Table 5:** Students’ levels of agreement with statements describing the course—‘Doctors’.

Statement Describing the Course (1 = Definitely Disagree; 5 = Definitely Agree)	*n*	Mean (SD)	M	Q1	Q3	Agree or Definitely Agree
During the course, I had the impression that I was talking with real patients.	30	4.30 (0.78)	4.5	4	5	80.00%
Scenarios realized during the course were too easy and did not constitute any challenge for me.	30	1.90 (0.75)	2	1	2	3.33%
Talking with the simulated patient, I was feeling like a real doctor.	30	4.03 (0.80)	4	4	5	76.67%
During the course, I felt the motivation to do the best I can to help the patient.	30	4.63 (0.55)	5	4	5	96.67%
The possibility to observe other students constituted an additional occasion to learn.	30	4.40 (0.84)	5	4	5	90.00%
The presence of other students as observers was not a problem for me.	30	3.93 (1.12)	4	3.25	5	73.33%
The presence of other students was troublesome and distracting.	30	2.23 (1.17)	2	1	3	23.33%
The feedback from simulated patients and other students made me realize things I did not notice before.	30	4.20 (0.87)	4	4	5	76.67%

*n*—number of respondents; M—median; Q1—lower quartile; Q3—upper quartile.

## Data Availability

The data used to support the findings in this study are available from the corresponding author upon request.
